# Supplementary material for: An emerging viral pathogen truncates population age structure in a European amphibian and may reduce population viability
Source: PeerJ. 2018 Nov 16;6:e5949. doi: 10.7717/peerj.5949 (PMC6241393; doi:10.7717/peerj.5949)
Supplement: Supplemental Information 1 — A comprehensive overview of the rationale and methodologies behind our populations projection matrix modelling analyses. [file peerj-06-5949-s001.docx]

An emerging viral pathogen truncates population age structure in a European amphibian and may reduce population viability.

# Supplementary Methods - Population Matrix Models.

Previous studies have shown that demographic shifts within a diseased population, or the change in vital rates which can bring such changes about, can reduce the stability and viability of populations (Saether & Bakke 2000) and may result in heightened population vulnerability to fluctuations within its environment (Rouyer et al. 2012; Scheele et al. 2017). To investigate whether the same might be true for UK *R. temporaria* populations affected by ranavirosis we used population projection matrix models. We firstly modeled hypothetical starting populations representative of both rananvirosis positive (truncated age structure) and disease-free populations based upon matrices populated with vital rates representative of both disease history population types. Secondly, we modelled a standard *R. temporaria* population (no age truncation) based upon matrices representing populations of both ranavirosis statuses and incorporating environmental instability. The following is a comprehensive explanation of our matrix modelling methodologies.

## “Disease-free” matrix construction and the impact of altered age structure on population dynamics

An initial female-based Leftkovich matrix was produced consisting of 11 stages; eggs, juvenile (sexually immature frogs) and adult age classes ranging from 2 to 10 years old. We populated our initial matrix using published vital rates for *R. temporaria* from Biek et al. (2002). Biek et al. use a three stage matrix and therefore assume a uniform maximum reproductive output for all sexually mature adult females of 650 female eggs per year. However, it has been shown that the reproductive output of *R. temporaria* is significantly influenced by body size (Gibbons & McCarthy 1986; but see Ryser 1996). In conjunction with the body size by age data generated in our field study, we expanded our matrix to include 9 adult age classes and adjusted the vital rates for fecundity to increase for every year post sexual maturity, starting at a rate of 250 eggs for two-year-old breeding animals and increasing by 50 eggs per year until an output of 650 eggs per year at adult age class 10. Since fecundity in our matrix is not uniform and female frogs from our dataset were found to mature at ages 2, 3 and 4 (Fig. S4; consistent with previous findings in *R. temporaria* e.g. Miaud et al. 1999), we accounted for variation in onset of sexual maturity by allowing juveniles to remain juveniles and to transition directly into adult classes 2, 3 or 4 with highest chance of reaching maturity at 3 years of age. See Table S8 for a numerical representation of the full initial matrix.

To investigate the consequence of an altered population age structure on population dynamics, independently of differing vital rates between populations of different disease history, we created an age distribution vector representative of each disease history group. We populated these vectors by calculating the proportion of individuals that were observed at populations of each disease history status that belonged to each age class and used those proportions as probabilities by which to weight a draw of 150 individual females that could belong to any age class. We used the projection function of the Popdemo package (Stott, Hodgson & Townley 2012) to project these two starting populations 20 years into the future based on our initial matrix which represented a population free from ranavirosis (additional parameters; standard.A = True, standard.vec = True).

## “Positive disease history” matrix construction and the impact of increased adult mortality on population dynamics

Deaths due to ranavirosis are known to occur annually in diseased populations of UK *R. temporaria* (Daszak et al. 1999; Teacher, Cunningham & Garner 2010), and annual breeding in permanent water bodies has been identified as a key ecological risk factor of disease (Hoverman et al. 2011). Additionally, our age structure data presented here is suggestive of increased adult mortality in populations with a positive history of ranavirosis. Therefore, to represent increasing annual chance of adult mortality due to ranavirosis at positive populations we created an additional matrix which reduced adult survival annually. No age-specific data are currently available on mortality rates of wild adult amphibians due to ranavirosis. We therefore modelled the population dynamics of a range of percentage decreases in annual survival in positive disease history populations (1-25% annual reduction). Using the same starting population vector (ranavirosis-free, no age truncation), we found no significant difference in population dynamics of any starting population based on decreasing likelihood of annual adult survival alone (Fig. S6, ANOVA; df = 6, F = 0.889, p = 0.505). In the absence of a robust estimate of decreased likelihood of annual adult survival due to ranavirosis, we selected a value of a 5% increased likelihood of mortality per year to represent theoretical ranavirosis-positive populations in further modelling. We used the projection function of the Popdemo package (Stott et al. 2012) to project our two starting population vectors 20 years into the future based on this “positive disease history” matrix (additional parameters; standard.A = True, standard.vec = True) and compared these projections to those based on our “disease-free” matrix.

## Elasticity analysis

To investigate which vital rates had the largest impact on the population dynamics of our two hypothetical populations we computed the elasticities of each matrix using the elasticity function of the pop.bio R package (Stubben & Milligan 2007). Matrix elasticities represent the proportional sensitivity of population growth dynamics to each matrix element, normalised so that the incomparable scales of survival and fecundity are removed (Groenendael, Kroon & Caswell 1988). Additionally, we compared the elasticities of our adjusted matrices to a third matrix constructed using the unadjusted vital rates published by Biek et al. 2002.

## Environmentally stochastic projection models

Previously, evidence has shown that an altered age structure can increase the susceptibility of a population to problems caused by a variable environment (Ohlberger et al. 2011; Rouyer et al. 2012; Scheele et al. 2016). To investigate the potential impact of environmental stochasticity on *R. temporaria* populations with and without persistent ranavirosis we created an additional set of transition matrices to represent differing ecological conditions. A well-known threat to the stability of amphibian populations is climate-induced reproductive failure (Shoo et al. 2011; Scheele et al. 2016), and in the UK, fluctuations in late winter/early spring night time temperatures can result in frost killing amphibian spawn. Low water temperatures have been identified as the principle threat to *R. temporaria* spawn in UK ponds (Beattie, Aston& Milner 1991), and are also associated with oomycete infections of amphibian egg masses, which can result in the total annual reproductive failure of a population (Beebee 1996). To examine the impact of such scenarios, we created alternate versions of both starting population matrices in which fecundity of all sexually mature adult classes was reduced to 0 (Tables S3 and S4).

To examine the additional impact of potentially recurrent mass mortality events due to ranavirosis, we created a further two matrices; one where the survival of all sexually mature adult classes was reduced to 20%, a biologically plausible but not extreme mortality estimate given that greater than 90% mortality has been observed during ranavirosis mortality events (Green, Converse & Schrader 2002), and a final matrix representing a catastrophic year in which both adult mass mortality and total reproductive failure occurred simultaneously (post-maturity adult survival of 20% and fecundity of 0, Tables S5 and S6). We used the stochastic projection function of the pop.bio package (Stubben & Milligan 2007) to project a *R. temporaria* population under the following stochastic scenarios;

A. A disease-free population with a 10% annual chance of reproductive failure;

B. A ranavirosis-positive population with a 10% annual chance of reproductive failure;

C. A ranavirosis-positive population with a 10% annual chance of either reproductive failure or an adult mass mortality event;

D. A ranavirosis-positive population with a 10% annual chance of either reproductive failure or an adult mass mortality event and a 5% annual chance of both challenges occurring simultaneously.

To ensure equality, all stochastic projections were initiated using the starting population vector of the disease-free populations. All projections ran for a duration of 100 years and for 5000 iterations. Based on our initial (non-stochastic) population projections we enforced a ceiling population carrying capacity of 200 sexually mature adult females summed across all age classes.

## Cited Literature

Beattie RC., Aston RJ., Milner AGP. 1991. A field study of fertilization and embryonic development in the common frog (Rana temporaria) with particular reference to acidity and temperature. *Journal of Applied Ecology* 28:346–357. DOI: 10.2307/2404134.

Beebee TJC (Trevor JC. 1996. *Ecology and conservation of amphibians*. Chapman & Hall.

Biek R., Funk WC., Maxell B a., Mills LS. 2002. What Is Missing from Insights Amphibian Decline Ecological Sensitivity Analysis. *Conservation Biology* 16:728–734. DOI: 10.1046/j.1523-1739.2002.00433.x.

Daszak P., Berger L., Cunningham A a., Hyatt a D., Green DE., Speare R. 1999. Emerging infectious diseases and amphibian population declines. *Emerging infectious diseases* 5:735–48. DOI: 10.3201/eid0506.990601.

Gibbons MM., McCarthy TK. 1986. The reproductive output of frogs Rana temporaria (L.) with particular reference to body size and age. *Journal of Zoology* 209:579–593.

Green DE., Converse K a., Schrader AK. 2002. Epizootiology of sixty-four amphibian morbidity and mortality events in the USA, 1996-2001. *Annals of the New York Academy of Sciences* 969:323–339. DOI: 10.1111/j.1749-6632.2002.tb04400.x.

Groenendael J van., Kroon H de., Caswell H. 1988. Projection matrices in population biology. *Trends in Ecology and Evolution* 3:264–269. DOI: 10.1016/0169-5347(88)90060-2.

Hoverman JT., Gray MJ., Haislip NA., Miller DL. 2011. Phylogeny, life history, and ecology contribute to differences in amphibian susceptibility to ranaviruses. *EcoHealth* 8:301–319. DOI: 10.1007/s10393-011-0717-7.

Miaud C., Guyétant R., Elmberg J. 1999. Variations in life-history traits in the common frog Rana temporaria (Amphibia: Anura): a literature review and new data from the French Alps. *Journal of Zoology* 249:61–73. DOI: 10.1017/S0952836999009061.

Ohlberger J., Langangen Ø., Edeline E., Moland Olsen E., Winfield IJ., Fletcher JM., Ben James J., Christian Stenseth N., Asbjørn L., Langangen ystein., Asbjorn Vollestad L. 2011. Pathogen-induced rapid evolution in a vertebrate life-history trait. *Vøllestad Source: Proceedings: Biological Sciences Proc. R. Soc. B* 278:35–41. DOI: 10.1098/rspb.2010.0960.

Rouyer T., Sadykov A., Ohlberger J., Stenseth NC. 2012. Does increasing mortality change the response of fish populations to environmental fluctuations? *Ecology Letters* 15:658–665. DOI: 10.1111/j.1461-0248.2012.01781.x.

Ryser J. 1996. Comparative life histories of a low and high-elevation population of the common frog Rana temporaria. *Amphibia-Reptilia* 27:183–195.

Saether B-E., Bakke O. 2000. Avian Life History Variation and Contribution of Demographic Traits to the Population Growth Rate. *Ecology* 81:642–653.

Scheele BC., Hunter DA., Banks SC., Pierson JC., Skerratt LF., Webb R., Driscoll DA. 2016. High adult mortality in disease-challenged frog populations increases vulnerability to drought. *Journal of Animal Ecology*:1–8. DOI: 10.1111/1365-2656.12569.

Scheele BC., Skerratt LF., Hunter DA., Banks SC., Pierson JC., Driscoll DA., Byrne PG., Berger L. 2017. Disease-associated change in an amphibian life-history trait. *Oecologia*. DOI: 10.1007/s00442-017-3911-7.

Shoo LP., Olson DH., Mcmenamin SK., Murray KA., Van Sluys M., Donnelly MA., Stratford D., Terhivuo J., Merino-Viteri A., Herbert SM., Bishop PJ., Corn PS., Dovey L., Griffiths RA., Lowe K., Mahony M., Mccallum H., Shuker JD., Simpkins C., Skerratt LF., Williams SE., Hero JM. 2011. Engineering a future for amphibians under climate change. *Journal of Applied Ecology* 48:487–492. DOI: 10.1111/j.1365-2664.2010.01942.x.

Stott I., Hodgson DJ., Townley S. 2012. Popdemo: provides tools for demographic modelling using project matrices.

Stubben C., Milligan B. 2007. Estimating and analyzing demographic models. *Journal Of Statistical Software* 22:1–23. DOI: 10.18637/jss.v022.i11.

Teacher AGF., Cunningham AA., Garner TWJ. 2010. Assessing the long-term impact of Ranavirus infection in wild common frog populations. *Animal Conservation* 13:514–522. DOI: 10.1111/j.1469-1795.2010.00373.x.
