# Supplementary figures and images for: An emerging viral pathogen truncates population age structure in a European amphibian and may reduce population viability
Source: PeerJ. 2018 Nov 16;6:e5949. doi: 10.7717/peerj.5949 (PMC6241393; doi:10.7717/peerj.5949)

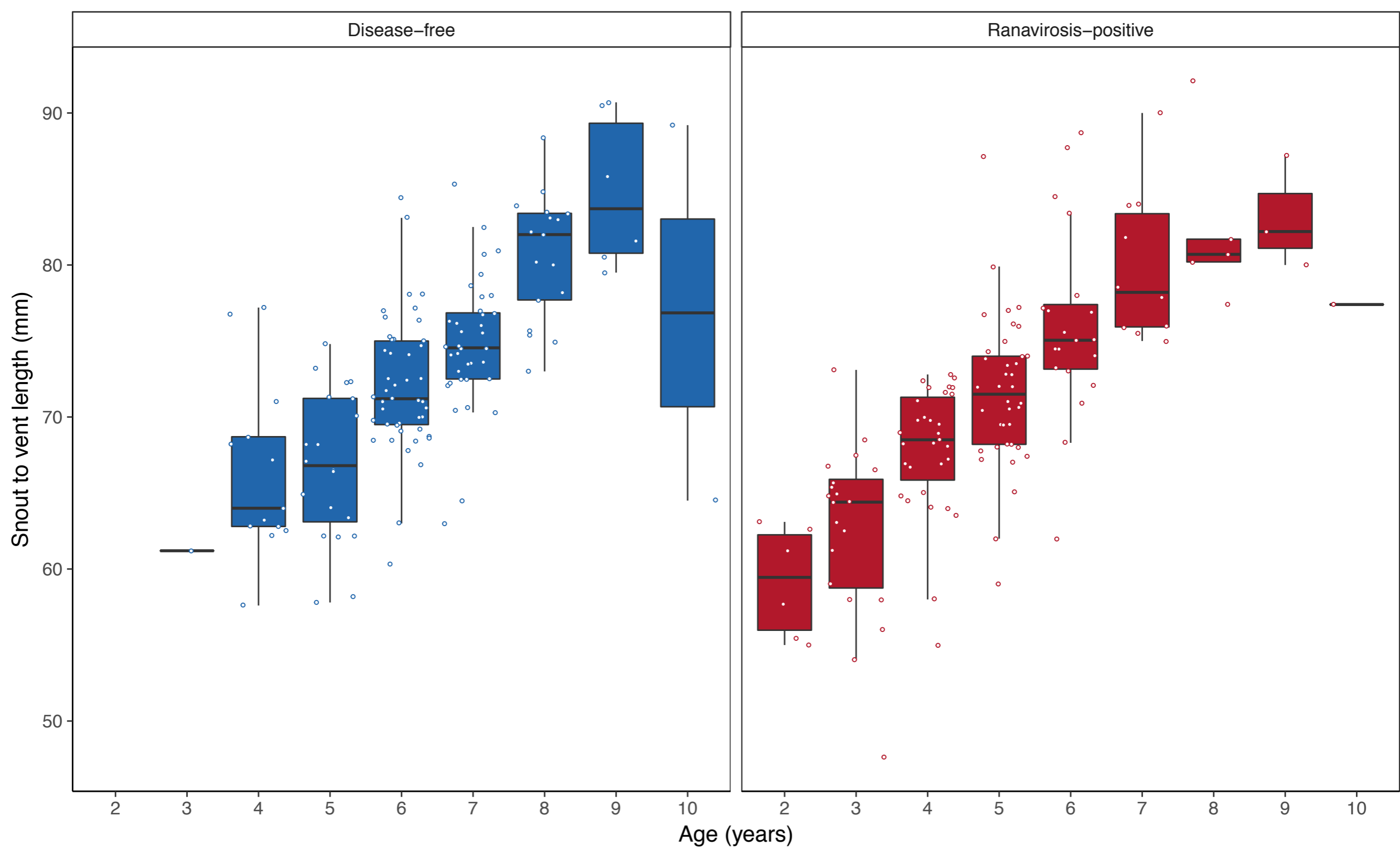

Supplement: Supplemental Information 2 — The relationship between snout to vent length (SVL) and age in R. temporaria from ranavirosis-positive and disease-free populations. We found no significant effect of disease history on the SVL of frogs. Our data does however show clear evidence of continued growth throughout life regardless of disease history. [file peerj-06-5949-s002.pdf]

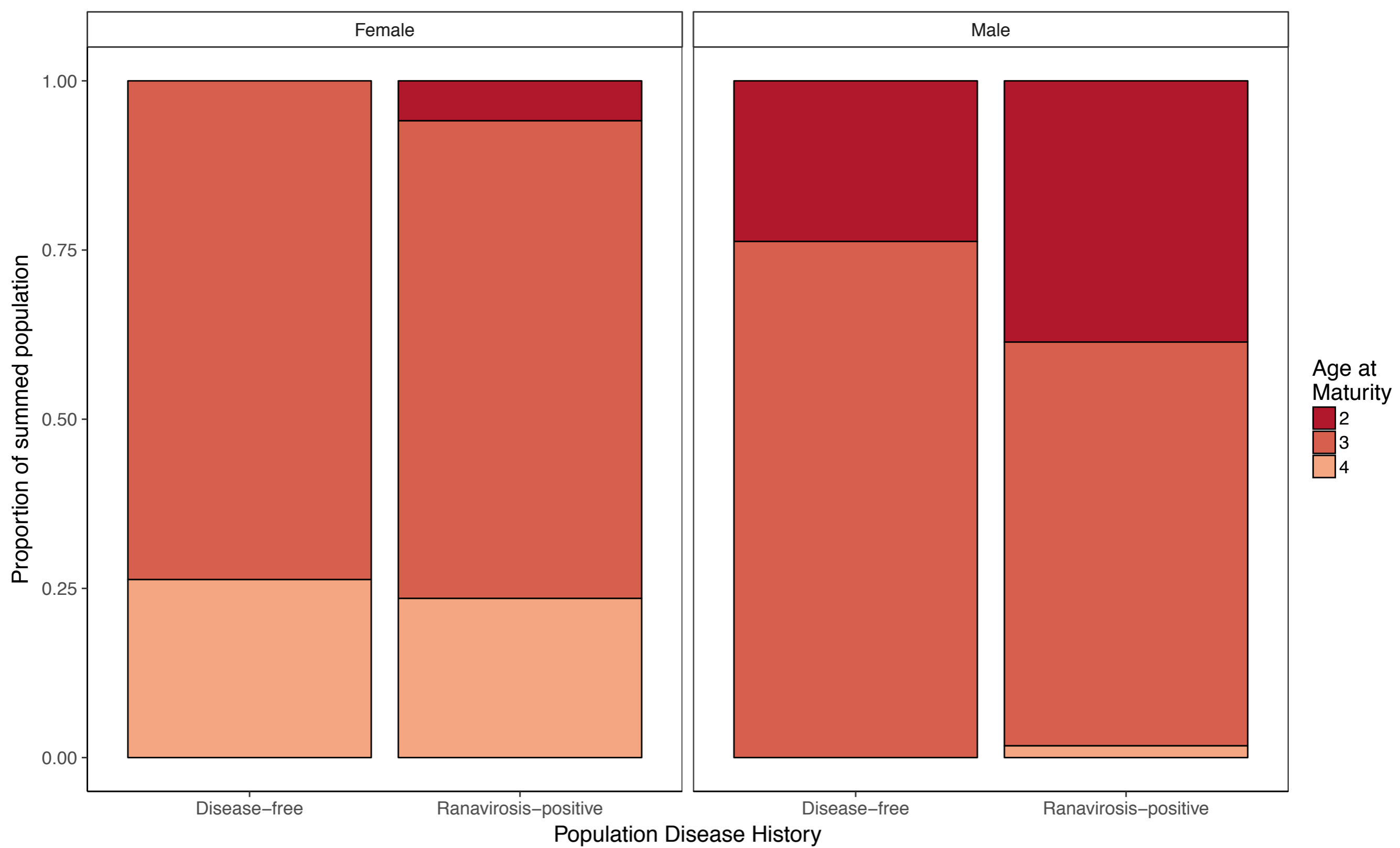

Supplement: Supplemental Information 3 — The proportion of individuals that reached sexual maturity at each possible age within the summed male and female populations of each disease history. Age at sexual maturity was calculated via skeletochronology. [file peerj-06-5949-s003.pdf]

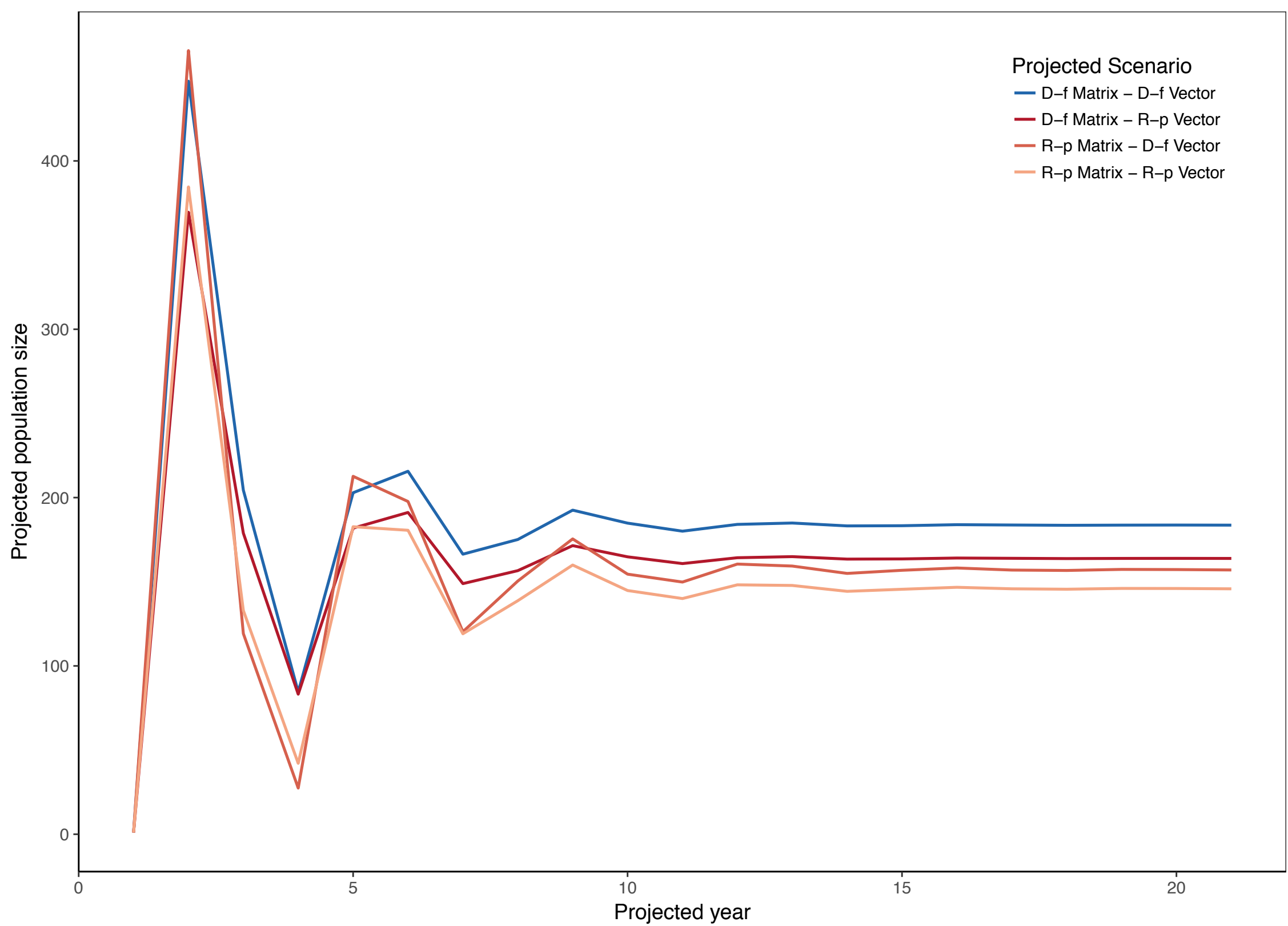

Supplement: Supplemental Information 4 — Projected population dynamics of R. temporaria populations modelled under different scenarios. Starting population vectors were created by weighted random sampling of 150 animals based on the proportions of each age class observed in populations of each disease history in our data. D-f = disease free and R-p = ranavirosis-positive. Our R-p matrix incorporated decreasing annual adult survival and our R-p starting population vector was age structure truncated. [file peerj-06-5949-s004.pdf]

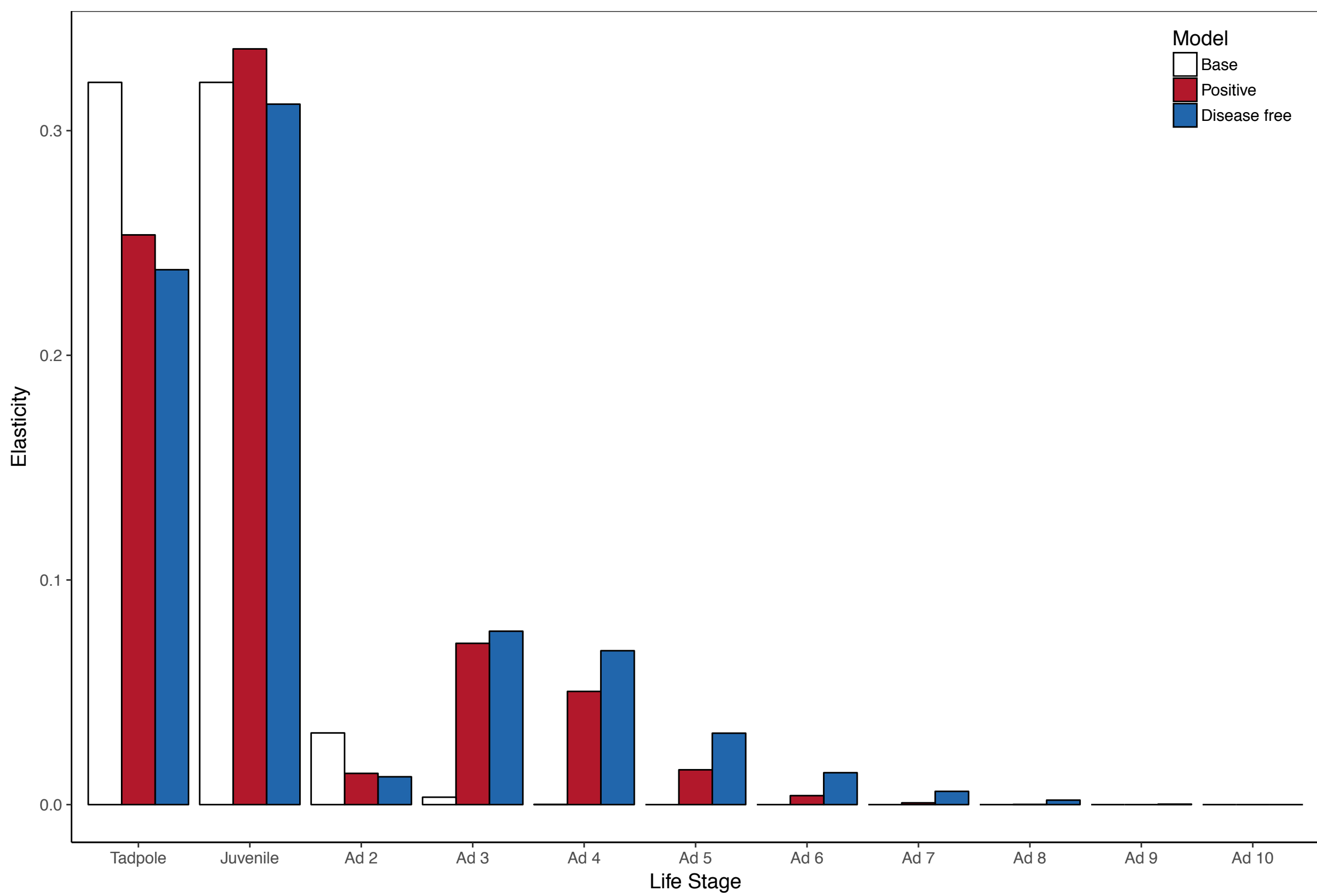

Supplement: Supplemental Information 5 — Computed elasticities of each survival element of our population projection matrices. Elasticities of our matrix which incorporated increasing adult mortality and therefore represented a population subjected to ongoing ranavirosis are shown in red, whilst those of our matrix which represented a disease-free population are shown in blue. For reference the elasticities of an extended matrix created using the unadjusted viral rates published by Biek et al. (2002) are shown in white. [file peerj-06-5949-s005.pdf]

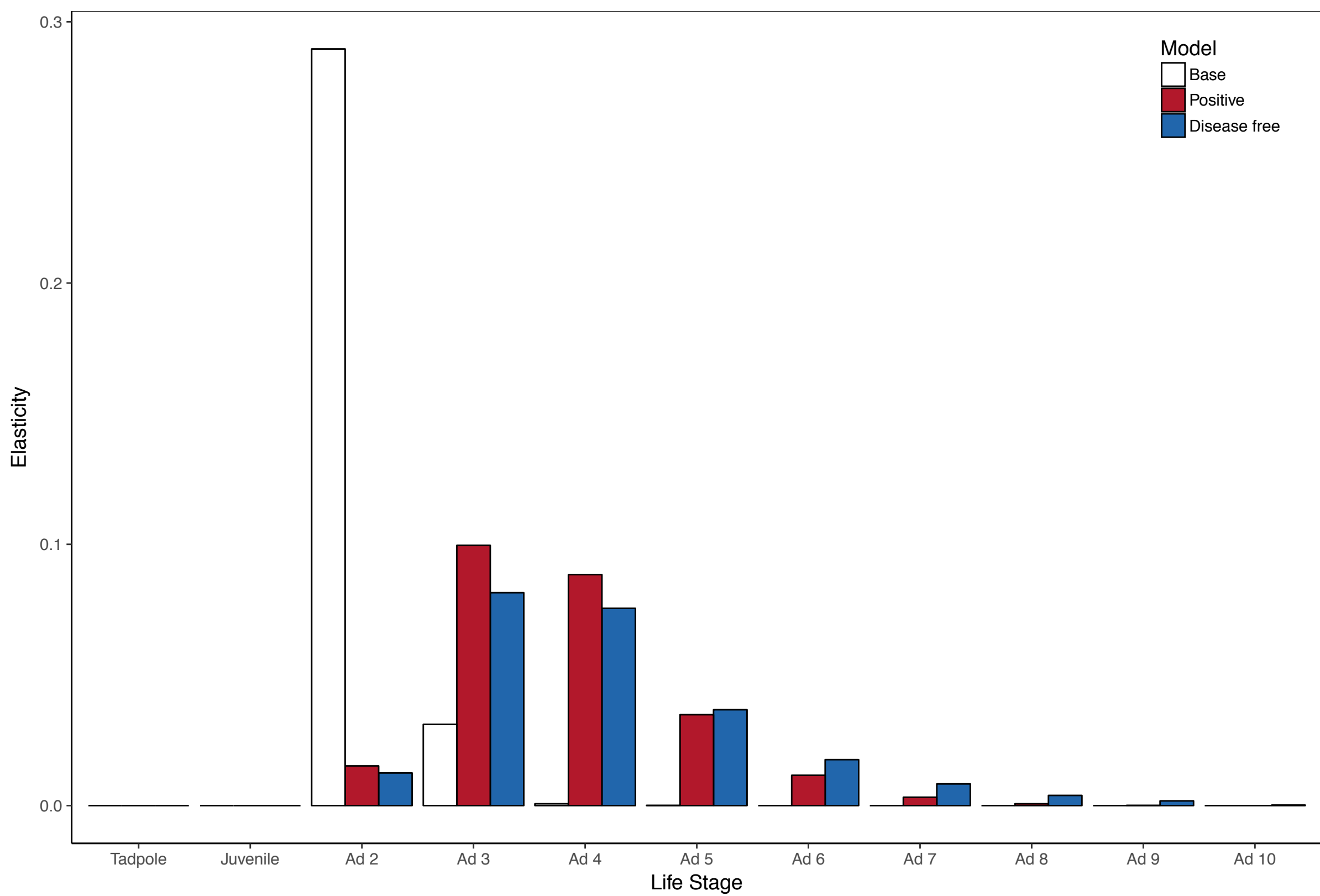

Supplement: Supplemental Information 6 — Computed elasticities of each fecundity element of our population projection matrices. Elasticities of our matrix which incorporated increasing adult mortality and therefore represented a population subjected to ongoing ranavirosis are shown in red, whilst those of our matrix which represented a disease-free population are shown in blue. For reference the elasticities of an extended matrix created using the unadjusted viral rates published by Biek et al. (2002) are shown in white. [file peerj-06-5949-s006.pdf]

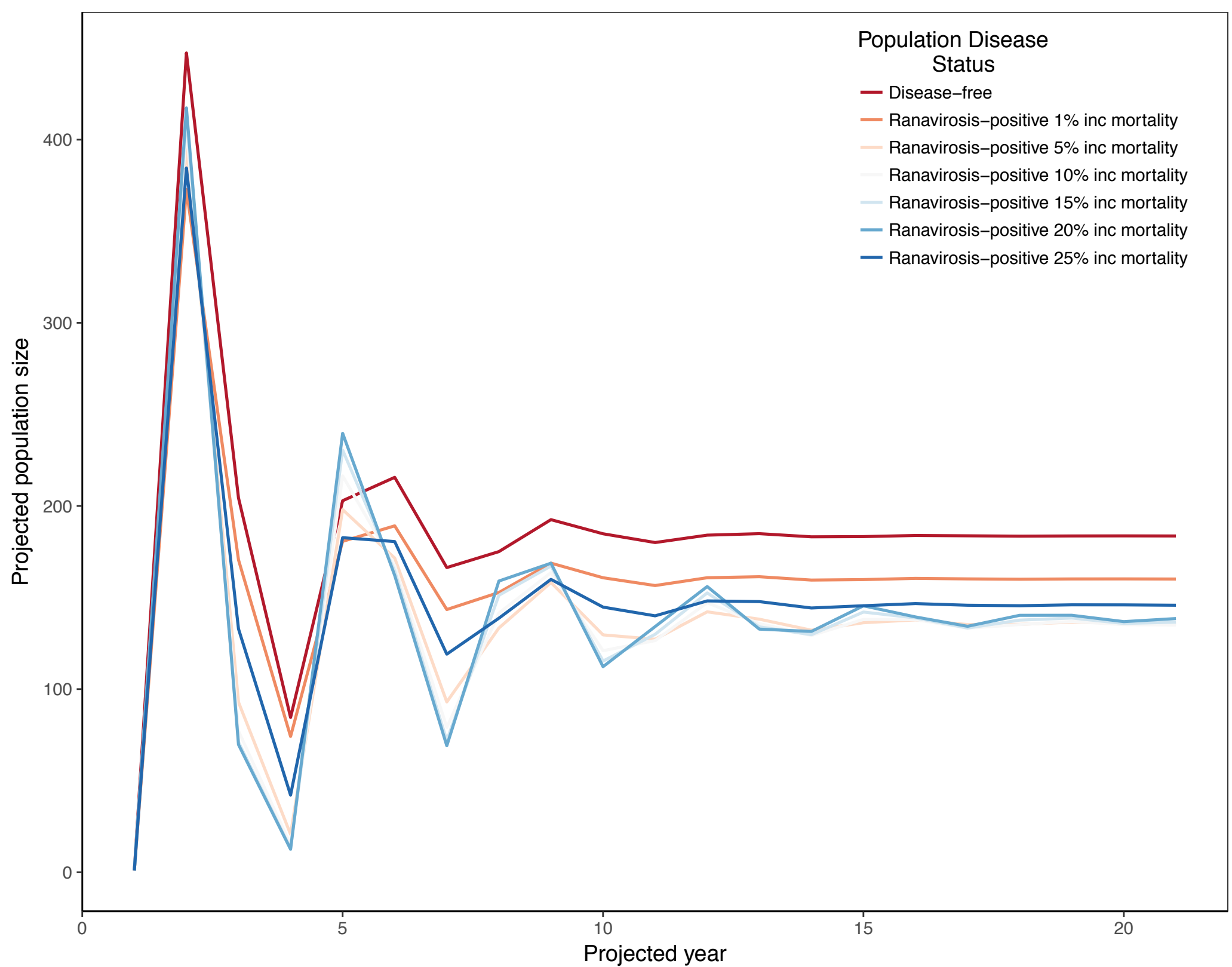

Supplement: Supplemental Information 7 — Population dynamics of theoretical disease-free populations and theoretical ranavirosis-positive populations modelled with an increasingly large annual percentage increase in adult mortality due disease. We found no significant difference between the population estimates through time based on varying this survival parameter alone (ANOVA; df = 6, F = 0.889, p = 0.505). In the absence of an empirically proven value for this parameter, we selected a value of 5% annual increase in mortality to represent the probabilistic increase of succumbing to ranavirosis with an increasing number of years returning to breed within the same infected population. [file peerj-06-5949-s007.pdf]
