# Supplementary material for: An emerging viral pathogen truncates population age structure in a European amphibian and may reduce population viability
Source: PeerJ. 2018 Nov 16;6:e5949. doi: 10.7717/peerj.5949 (PMC6241393; doi:10.7717/peerj.5949)
Supplement: Supplemental Information 8 — The number of R. temporaria sampled at each individual population. [file peerj-06-5949-s008.docx]

| **Population** | **Status** | **# of frogs sampled** |
| --- | --- | --- |
| Ealing | Positive | 46 |
| Poole | Positive | 43 |
| Southampton | Positive | 7 |
| Chessington | Positive | 30 |
| Tadworth | Positive | 21 |
| Mitcham | Disease Free | 61 |
| Folkington Corner | Disease Free | 4 |
| Palmer’s Green | Disease Free | 61 |
| Oxford | Disease Free | 26 |
| Witham | Disease Free | 5 |
