# Supplementary material for: An emerging viral pathogen truncates population age structure in a European amphibian and may reduce population viability
Source: PeerJ. 2018 Nov 16;6:e5949. doi: 10.7717/peerj.5949 (PMC6241393; doi:10.7717/peerj.5949)
Supplement: Supplemental Information 9 — Basal population matrix. Row one represents the fecundity of all life stages on the horizontal into the egg life stage, the number is equivalent to females produced per female. All other rows represent the proportional survival of each age class on the horizontal to the relevant age class on the vertical aspect of the matrix. [file peerj-06-5949-s009.docx]

|  | Egg | Juvenile | 2y | 3y | 4y | 5y | 6y | 7y | 8y | 9y | 10y |
| --- | --- | --- | --- | --- | --- | --- | --- | --- | --- | --- | --- |
| Egg | 0 | 0 | 250 | 300 | 350 | 400 | 450 | 500 | 550 | 600 | 650 |
| Juvenile | 0.019 | 0.25 | 0 | 0 | 0 | 0 | 0 | 0 | 0 | 0 | 0 |
| 2y | 0 | 0.01 | 0 | 0 | 0 | 0 | 0 | 0 | 0 | 0 | 0 |
| 3y | 0 | 0.05 | 0.45 | 0 | 0 | 0 | 0 | 0 | 0 | 0 | 0 |
| 4y | 0 | 0.02 | 0 | 0.45 | 0 | 0 | 0 | 0 | 0 | 0 | 0 |
| 5y | 0 | 0 | 0 | 0 | 0.45 | 0 | 0 | 0 | 0 | 0 | 0 |
| 6y | 0 | 0 | 0 | 0 | 0 | 0.45 | 0 | 0 | 0 | 0 | 0 |
| 7y | 0 | 0 | 0 | 0 | 0 | 0 | 0.45 | 0 | 0 | 0 | 0 |
| 8y | 0 | 0 | 0 | 0 | 0 | 0 | 0 | 0.45 | 0 | 0 | 0 |
| 9y | 0 | 0 | 0 | 0 | 0 | 0 | 0 | 0 | 0.45 | 0 | 0 |
| 10y | 0 | 0 | 0 | 0 | 0 | 0 | 0 | 0 | 0 | 0.1 | 0 |
